# Multimorbidity and Its Outcomes Among Patients Attending Psychiatric Care Settings: An Observational Study From Odisha, India

**DOI:** 10.3389/fpubh.2020.616480

**Published:** 2021-04-21

**Authors:** Sanghamitra Pati, Pranab Mahapatra, Rinshu Dwivedi, Ramesh Athe, Krushna Chandra Sahoo, Mousumi Samal, Ram Chandra Das, Mohammad Akhtar Hussain

**Affiliations:** ^1^Indian Council of Medical Research (ICMR)-Regional Medical Research Centre, Bhubaneswar, India; ^2^Department of Psychiatry, Kalinga Institute of Medical Sciences, Kalinga Institute of Industrial Technology (KIIT) University, Bhubaneswar, India; ^3^Department of Humanities and Science (Economics), Indian Institute of Information Technology, Tiruchirappalli, India; ^4^Department of Humanities and Science (Mathematics), Indian Institute of Information Technology, Dharwad, India; ^5^Menzies Institute for Medical Research, University of Tasmania, Hobart, TAS, Australia

**Keywords:** multimorbidity, physical-mental interface, health care utilization, polypharmacy, medical expenditure, OOPE, LMIC, psychiatric

## Abstract

**Background:** Multimorbidity, the presence of two or more chronic health conditions is linked to premature mortality among psychiatric patients since the presence of one can further complicate the management of either. Little research has focused on the magnitude and effect of multimorbidity among psychiatric patients in low-and middle-income settings. Our study, provides the first ever data on multimorbidity and its outcomes among patients attending psychiatric clinics in Odisha, India. It further explored whether multimorbidity was associated with higher medical expenditure and the interaction effect of psychiatric illness on this association.

**Methods:** This cross-sectional study included 500 adult patients presenting to the psychiatric clinic of a medical college hospital in Odisha over a period of 6 months (February 2019–July 2019). A validated structured questionnaire, “multimorbidity assessment questionnaire for psychiatric care” (MAQ-PsyC) was used for data collection. We used multinomial logistic model for the effect estimation. Odds ratios (OR) and 95% confidence intervals (CI) for high healthcare utilization and expenditure were calculated by number and pattern of multimorbidity. Data was analyzed by STATA 14.

**Results:** Half (50%) of the psychiatric outpatients had multimorbidity. The relative probabilities of having one additional condition were 5.3 times (RRR = 5.3; 95% CI: 2.3, 11.9) and multiple morbidities were 6.6 times (RRR = 6.6; 95%CI: 3.3, 13.1) higher for patients in 60+ age group. Healthcare utilization i.e., medication use and physician consultation was significantly higher for psychiatric conditions such as mood disorders, schizophrenia, schizotypal and delusional disorders, and for hypertension, cancer, diabetes, among somatic conditions. Out of pocket expenditure (OOPE) was found to be highest for laboratory investigations, followed by medicines and transport expenditure. Within psychiatric conditions, mood disorders incurred highest OOPE ($93.43) while hypertension was the most leading for OOPE in physical morbidities ($93.43). Psychiatric illnesses had a significant interaction effect on the association between multimorbidity and high medical expenditure (*P* = 0.001).

**Conclusion:** Multimorbidity is highly prevalent in psychiatric patients associated with significantly high healthcare utilization and medical expenditure. Such disproportionate effect of psychiatric multimorbidity on healthcare cost and use insinuates the need for stronger financial protection and tailor-made clinical decision making for these vulnerable patient subgroups.

## Introduction

Demographic transition accompanied by epidemiological shifts and changing lifestyle has led to a dramatic increase in chronic and non-communicable diseases (NCD) across low- and middle-income countries (LMICs) (1). According to the recent World Health Organization (WHO), Global Health Observatory (GHO) data, NCDs accounted for 71% of global deaths in LMICs in 2016 (1). Moreover, with the rapidly growing chronic conditions and increasing life-expectancies, multimorbidity (i.e., the presence of two or more chronic conditions) is being frequently observed in these populations (2–5). Multimorbidity is associated with an impaired functional capacity, increased health-care utilization, higher health care expenditure, marked lower quality of life, inferior mental health and ultimately, a higher risk for premature mortality (6–9). Given its common occurrence and substantial negative impact on individuals and health systems, multimorbidity is undoubtedly one of the most daunting public health challenges faced by health care providers and governments across the LMIC (10, 11). A recent global systematic review summarizing the community based studies on multimorbidity has reported the overall pooled prevalence of multimorbidity to be 29.7% (26.4–33.0%) in LMICs (12). Yet, most of the available reports from LMICs have their limitations predominantly being based on secondary analysis of population surveys or confined to elderly population using a list of few selected chronic conditions (13).

Within the multimorbid patient population, the presence of mental health conditions or psychiatric morbidities (e.g., depression, anxiety, etc.) accentuates the negative impact on clinical outcomes with disproportionately poorer quality of life and higher out-of-pocket expenditure (OOPE), higher medication use with and reduced life expectancy (14). A study of the impact of psychiatric conditions (depression/anxiety, substance abuse, psychotic, or bipolar disorder) on mortality among individuals with diabetes indicated that alcohol and drug abuse/dependence was associated with a 22% higher mortality (15). In fact, it is being promulgated that the increased mortality in psychiatric patients might be due to the concurrent presence of multimorbidity rather than the psychiatric illness itself (16, 17).

Interestingly, studies entailing multimorbidity in primary care patients, have highlighted the low prevalence of psychiatric illnesses like mood and psychotic disorders and substance abuse disorders (18, 19). Evidence suggests that chronic disease patients often do not report or seek treatment for their mental health conditions from primary healthcare system despite having increased contact and rather prefer to consult a psychiatrist or specialist for their mental illness (20). Thus, to assess the true magnitude of multimorbidity and its consequences among these patients, it is more prudent to include psychiatric care facility than general practice. However, when compared to their counterparts in rheumatology (21), cardiovascular (22) and HIV/AIDS (23), the impact of multimorbidity on health care resource use and health care cost in psychiatric patients have not been examined in-depth even in high income settings (24). A recent bibliometric analysis (25) had summarized global research trends and activities on multimorbidity, conspicuously, not a single study included or featured on multimorbidity among psychiatric patients from South Asia (12, 26, 27).

Within LMICs, India, the largest demography contributes to a considerable share of NCD globally, and thus, multimorbidity is a frequently encountered phenomenon in healthcare settings (19, 27). Our own previous study, the first ever to assess multimorbidity in primary care, has estimated the prevalence to be one third with considerable worsening of quality of life and associated with depression and high health care utilization (28, 29). At the same time, according to the National Mental Health Survey, there are nearly 197.3 million people living with mental disorders in India which includes 45.7 million people with depressive and 44.9 million with anxiety disorders (30). Given the rising burden of psychiatric disorders along with escalating prevalence of chronic conditions, there is a clear need to better understand the magnitude, determinants and consequences of multimorbidity as a whole in the psychiatric patient population, in order to better organize and provide care, as well as to develop appropriate research models (31). While progress has been made, most studies to date have only examined the co-occurrence of psychiatric illness (either anxiety or depression) with a single comorbid physical health condition (32) or the coexistence of two psychiatric disorders (33). This constitutes a knowledge disconnect, since for psychiatrists, understanding the complex role of multimorbidity is indispensable to provide safe, efficient, and optimal care for patients although little is known about the impact these diseases have (34).

With an aim to address the extant research gap, the present study was undertaken to generate first ever evidence on multimorbidity in psychiatric care settings in India. Our aim was to estimate the prevalence of multimorbidity, assess the outcomes (in terms of hospitalization, number of medications, physician consultation, healthcare expenditure) across different conditions within multimorbidity and identify the correlates (age, gender, religion, education, occupation, and social security). We expect our findings to yield new insights to delineate high-risk psychiatric patient subgroups, which could preferentially benefit from tailored preventative and therapeutic strategies adapted for multimorbidity and enhance the health outcomes at the individual, communities and health systems level. Further the findings would be of significant importance for the currently rolled out National Health Assurance Program for Universal Health Coverage in India (35).

## Materials and Methods

### Study Design, Setting, and Participants

This observational study was conducted among adult patients (18 years and above) attending the psychiatric outpatient's department (OPD) of the Kalinga Institute of Medical Sciences (KIMS) –tertiary health care and teaching hospital in the state of Odisha, India, from February 2019 to July 2019. Since we did not have any data on the prevalence of psychiatric multimorbidity, and given the study duration of 6 months, we decided to fix the sample size of 500 patients through an initial careful assessment of the average outpatient attendance and the number of patients coming for the first time to the facility per day. From our pilot observations, we found that at least 40 to 50 adult patients were attending the psychiatry OPD per day, out of which, nearly 10 were first time attendees. Thus, we decided to include all first time visiting patients on every alternate working day (about 12 days per month) for 6 months, which made up to 500. This allowed us to accommodate non-response, ineligibility, and incomplete interviews if any.

Those who provided the consent were interviewed only after the consultation with the psychiatrists to avoid any disturbance to the hospital patient management system and delays. Also, the exit interviews helped us to record the diagnosis in detail by going through the prescriptions. To avoid duplication each patient was given unique identification number and those who have already been interviewed previously under the present study were excluded during follow up consultation. Patients too ill to participate, those with insufficient cognitive ability to complete the interview and those with debilitating physical and mental conditions and not willing to participate were excluded from the study.

### Data Collection

A structured multimorbidity assessment questionnaire for psychiatric care (MAQ-PsyC) was employed for collecting data. This tool MAQ-PsyC is an adapted version of our previously used multimorbidity assessment questionnaire for primary care (MAQ-PC). We had developed and validated MAQ-PC through an iterative process and demonstrated a good concordance with clinical records and physician prescriptions (36). A tablet-based Epi-info format was designed to capture the information through MAQ-PsyC. The MAQ-PsyC has three sections: sociodemographic data, multimorbidity (physical and psychiatric) assessment, and outcomes (health care utilization and health care expenditure).

The sociodemographic section MAQ-PsyC included information on participant's age, gender, marital status (currently married or not), caste (Scheduled castes (SCs)/Scheduled Tribes (STs), General (Other categories) religion (Hindu, Non-Hindu (including Muslims, Christians, and others), place of residence (rural/urban); the level of education (including not educated, up to the primary, up to high/senior secondary, graduation and above), type of occupation—regular wage or salaried, employed in the organized sector, self-employed, casual, others (housewives and students), and unemployed, eligibility for social security (37).

Multimorbidity assessment section of MAQ-PsyC comprised psychiatric morbidities assessment, which were diagnosed by psychiatrist using International Classification of Diseases (ICD 10-DSR) (38–40) and the physical morbidity assessment included our previous list of self-reported doctor diagnosed chronic conditions namely—arthritis, diabetes, hypertension, chronic lung disease, acid peptic disease (APD), musculoskeletal disorders, heart disease and stroke, neurological, visual impairment, hearing impairment, cancer and tumor, chronic-kidney-diseases, thyroid diseases, disability, and certain chronic infectious diseases such as tuberculosis, HIV, and filariasis. These conditions were self-reported doctor diagnosed and cross-validated from the patient prescriptions and medicine wrappers (27).

We also elicited information on health outcomes namely healthcare utilization (the number of prescribed medications, the number of drugs currently on, hospitalization in the facility and duration of hospital stays, visit to public and private providers) and health care expenditure in form of OOPE. Detailed information on expenditure on medicines, healthcare-related travel, and laboratory/diagnostics was obtained (27). The recall periods for reported conditions and other health outcomes were for the last 12 months. The expenditure on medication was recorded for 30 days before the interview dates, while for the expenditure on travel and laboratory investigations, it was recorded for the last 6 months. The respondents were asked; “how many times in the last 6 months have you visited (traveled) a health care facility?,” and “how much money on an average have you spent in the last 6 months on laboratory investigations and tests?” For the present study, all the recorded information was converted into 12 months by following the approach to measuring OOPE for healthcare payments from Wagstaff and Doorslaer (41).

Interviews were conducted by two trained nurses those who had prior experience in data collection in multimorbidity as well as psychiatric disorders. They were well-versed with local language and patient history taking. Each interview spanned from 20 to 30 min.

Ethical approval for the study was obtained from The Institutional Ethics Committee of KIMS, Bhubaneswar, Odisha (Vide no. KIMS/KIIT/IEC/204/2018 dated 14.12.2018). Written informed consent was obtained from all participants, and necessary steps were taken to ensure confidentiality and anonymity of patients. In case of the inability to provide the necessary information by some of the patients, their caregivers were requested to provide relevant information. Approval of the hospital and outpatient department in charge was obtained prior to data collection.

### Statistical Analysis

For analysis, we defined multimorbidity as the presence of two or more chronic conditions (42). Considering age as one of the important predictors of multimorbidity and its related health outcome (29, 42), we decided to perform all analyses based on age-stratification. Age-stratified descriptive statistics were computed to assess the socio-economic and demographic profile of participants as well as the age wise distribution of major psychiatric and somatic conditions. We also explored the distribution of study participants according to the presence of physical morbidities. Further we examined the pattern of healthcare utilization among participants and the association between the morbidity conditions (physical or psychiatric or both) and healthcare utilization. Variations in OOPE [In INR (95% CI)] by morbidity conditions among the sampled population were also estimated.

The multinomial logistic model (MLM) was used as it allows the researchers to examine strategic choices with multiple outcomes (43, 44). We estimated the Relative Risk Ratios (RRR) with 95% confidence intervals (CI) for the likelihood of occurrence of multiple comorbid conditions as per the socio-economic covariates of the patients. The RRR was interpreted as percentage increase or decrease in relative probability of being in one group compared to the reference group. In addition, we employed multiple Generalized Linear Regression Model (GLM) to explore the effect of various socio-economic covariates on the level of OOPE among people living with psychiatric illnesses (45). Our outcome variable OOPE was usually non-parametric and positively skewed with influential outliers. GLM can flexibly handle the skewed datasets and reduce the problem of outcome transformation. We have employed GLM with gamma distribution and log link function to examine the various determinants of OOPE (46, 47). All statistical analyses were performed using STATA 14 (Stata Corp, College Station, Texas).

## Results

The mean age of the study participants was 39.8 years (Standard deviation, SD ± 15.3). Out of the total 500 participants, 261 (52%) were male. Age-stratified distribution of study participants and their demographic characteristics are presented in Table 1. More than half (55%) of the study participants were of the younger age group (18–39 years) followed by 40–49 years (32%) and 60+ years (13%). Overall, half of the study participants used to live in rural areas, more than two-thirds were married and about three-fourth were not covered via any social security scheme. Furthermore, more than one-third of the study participants were either housewives or students and were educated up to graduation or above at the time of the interview.

**Table 1:** Characteristic of study participants enrolled in the study by age groups.

	**Age groups (years)**			
**Covariates**	**18–39 years** **(*n* = 273)**	**40–59 years** **(*n* = 162)**	**60+ years** **(*n* = 65)**	**Total** **(500)**
**Gender**				
Female	125 (45.8)	84 (51.8)	30 (46.2)	239 (47.8)
Male	148 (54.2)	78 (48.1)	35 (53.9)	261 (52.2)
**Residence**				
Urban	139 (50.9)	76 (46.9)	38 (58.5)	253 (50.6)
Rural	134 (49.1)	86 (53.1)	27 (41.5)	247 (49.4)
**Caste**				
Schedule caste/scheduled tribe	42 (15.4)	15 (9.3)	7 (10.8)	64 (12.8)
General	231 (84.6)	147 (90.7)	58 (89.3)	436 (87.2)
**Religion**				
Hindu	260 (95.2)	155 (95.7)	61 (93.9)	476 (95.2)
Non-Hindu	13 (04.8)	7 (04.3)	4 (6.2)	24 (4.8)
**Marital status**				
Currently not married	139 (50.9)	12 (07.4)	11 (16.9)	162 (32.4)
Currently married	134 (49.1)	150 (92.6)	54 (83.1)	338 (67.6)
**Education**				
Not educated	14 (05.1)	24 (14.8)	12 (18.5)	50 (10.0)
Up to primary	34 (12.5)	34 (20.9)	18 (27.7)	886 (17.2)
Up to high/senior secondary	80 (29.3)	51 (31.5)	13 (20.0)	144 (28.8)
Graduation and above	145 (53.1)	53 (32.7)	22 (33.9)	220 (44.0)
**Occupation**				
Regular/salaried/business	68 (24.9)	39 (24.1)	14 (21.5)	121 (24.2)
Casual laborers	33 (12.1)	27 (16.7)	8 (12.3)	68 (13.6)
Others^*^	115 (42.1)	73 (45.1)	26 (40.0)	214 (42.8)
Unemployment	57 (20.9)	23 (14.2)	17 (26.2)	97 (19.4)
**Eligible for social security**				
No	194 (71.1)	123 (75.9)	54 (83.1)	371 (74.2)
Yes	79 (28.94)	39 (24.1)	11 (16.9)	129 (25.8)

* *Represents students and housewives*.

**Figure 1:**
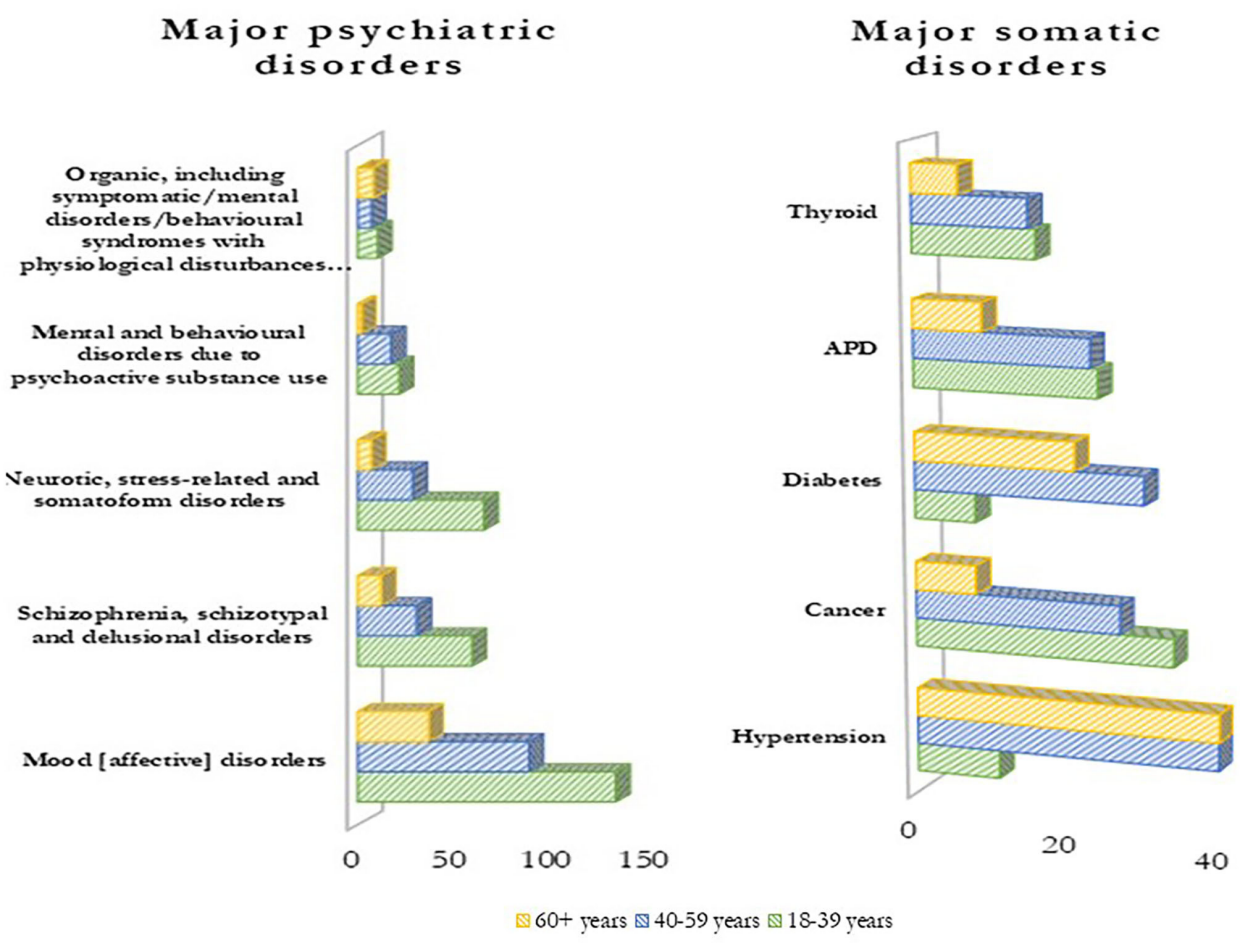
Distribution of major psychiatric and somatic disorders by age groups.

Figure 1 indicates the major psychiatric and physical morbidities by age groups. Among the psychiatric conditions, mood affective disorders (henceforth termed as mood disorders) were most common (51%) followed by schizophrenia, schizotypal and delusional disorders (20%), and neurotic, stress-related, and somatoform disorders (20%). Among physical morbidities, hypertension (18%), cancer (14%), and diabetes (12%) were the most commonly reported. Overall, the frequency of both psychiatric and physical morbidities was higher in younger patients (18–39 years) except for hypertension and diabetes, which were more prevalent among older age groups (more than 40 years). Distribution of study participants according to the number of physical morbidities among the study participants is presented in Table 2.

**Table 2:** Distribution of study participants according to the presence of physical comorbidities (*N* = 500).

**Covariates**	**No-comorbidity *n* = 250 (50.0%)**	**One comorbidity *n* = 137 (27.4%)**	**2+comorbidities *n* = 113 (22.6%)**	**Chi-square value**	***p*-value**
**Age**					
18–39	64.9	22.3	12.8	90.54	0.0
40–59	37	33.3	29.7		
60+	20	33.8	46.2		
**Gender**					
Female	48.5	29.7	21.8	4.06	0.5
Male	51.3	25.3	23.4		
**Residence**					
Urban	44.7	30.4	24.9	9.17	0.1
Rural	55.5	24.3	20.2		
**Caste**					
Schedule caste/tribe	57.8	28.1	14.1	7.07	0.2
General	48.9	27.3	23.9		
**Religion**					
Hindu	50.6	27.5	21.8	12.84	0.0
Non-Hindu	37.5	25	37.5		
**Marital status**					
Unmarried/divorced/widowed	63.6	20.4	16	22.21	0.0
Currently married	43.5	30.8	25.7		
**Education**					
Not educated	44.8	34	22	15.75	0.3
Up to primary	57	24.4	18.6		
Up to high/senior secondary	54.2	23.6	22.2		
Graduation and above	45.9	29.5	24.5		
**Occupation**					
Regular/salaried/business	41.3	35.5	23.1	32.9	0.0
Casual laborers	36.8	35.3	27.9		
Others	56.1	20.6	23.3		
Unemployment	56.7	26.8	16.5		
**Eligible for social security**					
No	45.8	28.6	25.6	13.03	0.0
Yes	62	24	14		

Half of the psychiatric patients had multimorbidity and 45.2% (*n* = 113) had more than two conditions. Age (chi-square; 90.5) and occupation (chi-square; 32.9) were significantly associated with the presence of physical morbidities, along with marital status, education, and coverage under social security for the respondents.

On multinomial logistic regression, higher age patients and non-Hindu religion were more likely to be associated with having multiple morbidities (Table 3).

**Table 3:** Results from Multinomial logistic regression analysis.

**Multinomial logit model with the base outcome (Only psychiatric)**						
**Covariates**	One comorbidity^*^			Multiple-comorbidities^**^		
	**RRR**	**CI (95%)**		**RRR**	**CI (95%)**	
**Age (Ref. 18-39 years)**						
40–59	2.5	1.5	4.4	2.9	1.6	5.1
60+	5.3	2.3	11.9	6.6	3.3	13.1
**Gender (Ref. Female)**						
Male	0.8	0.5	1.3	1.1	0.7	1.7
**Residence (Ref. Urban)**						
Rural	0.8	0.5	1.3	1.0	0.6	1.7
**Caste (Ref. SC/ST)**						
General	1.0	0.5	2.1	2.2	0.9	5.5
**Religion (Ref. Hindu)**						
Non-Hindu	1.4	0.4	4.8	3.7	1.2	10.9
**Marital Status (Ref. Unmarried/divorced/widowed)**						
Currently married	1.5	0.9	2.6	1.1	0.6	1.9
**Education (Ref. Not educated)**						
Up to primary	0.5	0.2	1.3	0.9	0.3	2.1
Up to higher/senior secondary	0.7	0.3	1.6	1.4	0.5	3.2
Graduation and above	1.1	0.5	2.7	1.6	0.6	3.8
**Occupation (Ref. Regular/salaried/business)**						
Casual laborers	0.9	0.5	1.9	1.1	0.6	2.5
Others	0.3	0.2	0.6	0.9	0.6	1.7
Unemployment	0.4	0.3	0.9	0.6	0.3	1.3
**Social security (Ref. No)**						
Yes	0.8	0.8	1.3	0.6	0.3	1.1

* *includes psychiatric and one somatic*;

** *includes psychiatric and two or more somatic*.

Increasing age was strongly and positively associated with multimorbidity among patients. The relative probabilities of having multimorbidity were 2.5 times (RRR = 2.5; 95% CI: 1.4–4.4) and 5.3 times (RRR = 5.3; 95% CI: 2.3, 11.9) higher for patients in 40–59 and 60+ age group compared to the 18–39 years age group patients.; Similarly, the relative probabilities of having multiple conditions were 2.9 times (RRR = 2.9; 95% CI: 1.6, 5.1) and 6.6 times (RRR = 6.6; 95% CI: 3.3, 13.1) higher for 40–59 year and 60+ age group. It was observed that religion does have some impact, as population which belong to the non-Hindu religion have recorded higher multiple morbidities (RRR = 3.7; 95% CI: 1.2, 10.9). Similarly, the type of occupation was also associated with multimorbidity, regular/salaried/business population, and others (includes students and housewives; RRR = 0.4; 95% CI: 0.2, 0.7) were found having 63 percent, and unemployed (RRR = 0.5; 95% CI: 0.3, 0.9) had around 50 percent significantly lower number of conditions. Moreover, caste affiliations and educational attainment have some association with the occurrence of multimorbidity, though the evidence was not statistically significant.

**Figure 2:**
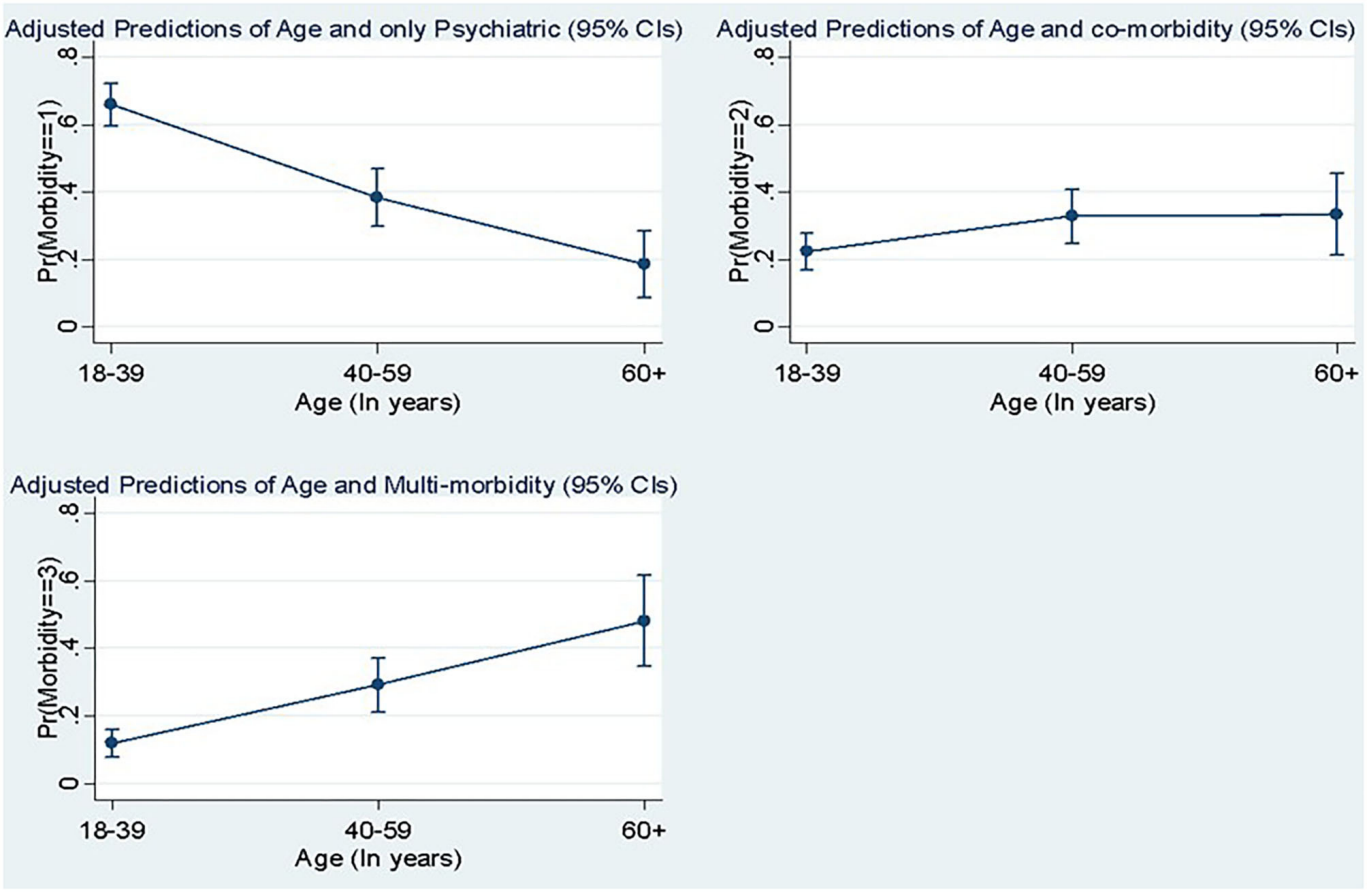
Predicted probabilities of the number of comorbidities with age.

Predicted probabilities of the number of morbidities along with age are presented in Figure 2, which reveals that except the younger age group who have higher psychiatric conditions, increasing age was positively and significantly associated with the number of conditions and multimorbidity.

Table 4 shows the pattern of healthcare utilization among the respondents by age. More than two-thirds of the patients have never sought any institutional care. The majority of the respondents (74%) never visited any public healthcare provider rather sought care from the private facilities irrespective of the age group. About 90 percent of the sampled population has visited the private providers, where the population in the age bracket of 40–59 years have recorded highest visits to the private providers (90.7%), followed by other groups. The maximum duration of the stay in any facility for the patients ranged between 0 and 3 days. Regarding medicine consumption, polypharmacy was seen in almost eighty percent (65 + 20%) of the patients. Almost seventy percent of patients in the age bracket of 18–39 years were consuming 1–5 medicines. About half of the elderly (60+ years) populations were consuming more than six medicines.

**Table 4:** Pattern of healthcare utilization among the respondents by age.

	**Age groups (years)**					
**Covariates**	**18–39 years** **(*n* = 273)**	**40–59 years** **(*n* = 162)**	**60+ years** **(*n* = 65)**	**Total** **(500)**	**Chi-square**	***p*-value**
**Number of in-patient hospitalization (Public/Private)**						
Never	182 (66.8)	118 (72.8)	44 (67.7)	344 (68.8)	1.8	0.3
Hospitalized (≥1)	91 (33.3)	44 (27.1)	21 (32.3)	156 (31.2)		
**Outpatient visit to public providers**						
Never	198 (72.5)	116 (71.6)	54 (83.0)	368 (73.6)	3.4	0.1
≥1	75 (27.5)	46 (28.4)	11 (16.9)	132 (26.4)		
**Outpatient visit to private providers**						
Never	28 (10.3)	15 (9.7)	7 (10.8)	50 (10.0)	0.1	0.9
≥1	245 (89.7)	147 (90.7)	58 (89.2)	450 (90.0)		
**Duration of stay in the facility**						
0-3 days	216 (79.1)	127 (78.4)	53 (81.5)	396 (79.2)	0.2	0.8
4+ days	57 (20.9)	35 (21.6)	12 (18.5)	104 (20.8)		
**Medicine consumption practices (Polypharmacy)**						
0 medicine	57 (20.9)	15 (9.3)	5 (7.7)	77 (15.4)	57.0	0.0
1-5 medicines	190 (69.6)	105 (64.8)	30 (46.6)	325 (65.0)		
6+ medicines	26 (9.5)	42 (25.9)	30 (46.2)	98 (19.6)		

Association between multimorbidity (psychiatric + physical) and healthcare utilization is depicted in Table 5. It was observed that for mood disorders about 32% of patients were admitted and have stayed in the hospital for an average duration of 4 days. The majority of them had visited private providers (91%), and were taking more than five medicines per day. Almost all the patients having schizophrenia, schizotypal and delusional disorders, have availed private healthcare multiple times, and about three-fourths of them were taking more than one (2–5) medicines. The majority of the patients having neurotic, stress-related, and somatoform disorders have availed private care (91%), and two-thirds were taking more than one medicine. The hospitalization rate was higher for patients with mental and behavioral disorders due to psychoactive substance use, and about 73% had availed private care. Further, it was observed that hospitalization for the organic, including symptomatic/mental disorders/behavioral syndromes with physiological disturbances and physical factors/mental retardation, 88% of patients have sought care from private providers one or more times and 66% were consuming 1–5 medicines.

**Table 5:** Association between the morbidity conditions (Psychiatric + Somatic) and healthcare utilization.

**Covariates**	***N***	**Hospitalization in the facility**		**Chi-square value**	***p*-value**	**Visit public providers**		**Chi-square value**	***p*-value**	**Visit private providers**		**Chi-square value**	***p*-value**	**Duration of stay in the facility**		**Chi-square value**	***p*-value**	**Polypharmacy**			**Chi-square value**	***p*-value**
		**No (344)**	**Yes (156)**			**Never (368)**	**≥One (132)**			**Never (50)**	**≥One (450)**			**0-3 days (396)**	**4+ days (104)**			**No medicine (77)**	**1–5 medicines (325)**	**6+ medicines (98)**		
**Psychiatric conditions**																						
Mood disorders	No (246)	171 (69.5)	75 (30.5)	0.1	0.7	179 (72.8)	67 (27.2)	0.2	0.6	28 (11.4)	218 (88.6)	1.0	0.3	194 (78.9)	52 (21.1)	0.0	0.8	43 (17.5)	167 (67.9)	36 (14.6)	8.1	0.0
	Yes (254)	173 (68.1)	81 (31.9)			189 (74.4)	65 (25.6)			22 (8.7)	232 (91.4)			202 (79.5)	52 (20.5)			34 (13.4)	158 (62.2)	62 (24.4)		
Schizophrenia, schizotypal, and delusional disorders	No (400)	272 (68.0)	128 (32.0)	0.6	0.4	293 (73.3)	107 (26.7)	0.1	0.7	42 (10.5)	358 (89.5)	0.6	0.4	319 (79.7)	81 (20.3)	0.4	0.5	65 (16.3)	250 (62.5)	85 (21.3)	5.6	0.0
	Yes (100)	72 (72.0)	28 (28.0)			75 (75.0)	25 (25.0)			8 (8.0)	92 (92.0)			77 (77.0)	23 (23.0)			12 (12.0)	75 (75.0)	13 (13.0)		
Neurotic, stress-related, and somatoform disorders	No (401)	267 (66.6)	134 (33.5)	4.6	0.0	302 (73.3)	99 (24.7)	3.1	0.0	41 (10.2)	360 (89.8)	0.1	0.7	307 (76.6)	94 (23.4)	8.6	0.0	57 (14.2)	259 (64.6)	85 (21.2)	4.5	0.1
	Yes (99)	77 (77.8)	22 (22.2)			66 (66.7)	33 (33.3)			9 (9.1)	90 (90.9)			89 (89.9)	10 (10.1)			20 (20.2)	66 (66.6)	13 (13.1)		
Mental and behavioral disorders due to psychoactive substance use	No (460)	323 (70.2)	137 (29.8)	5.4	0.0	338 (73.3)	122 (26.5)	0.0	0.8	39 (8.5)	421 (91.5)	14.8	0.0	373 (81.1)	87 (18.9)	12.4	0.0	65 (14.1)	307 (66.7)	88 (19.2)	9.3	0.0
	Yes (40)	21 (52.5)	19 (47.5)			30 (75.0)	10 (25.0)			11 (27.5)	29 (72.5)			23 (57.5)	17 (42.5)			12 (30.0)	18 (45.0)	10 (25.0)		
Organic, including symptomatic/mental disorders/behavioral syndromes with physiological disturbances and physical factors/Mental retardation	No (475)	329 (69.3)	146 (30.7)	1.0	0.3	346 (72.8)	129 (27.2)	2.8	0.0	47 (9.9)	428 (90.1)	0.1	0.7	376 (79.2)	99 (20.8)	0.0	0.9	73 (15.4)	310 (65.3)	92 (19.3)	0.4	0.8
	Yes (25)	15 (60.0)	10 (40.0)			22 (88.0)	3 (12.0)			3 (!2.0)	22 (88.0)			20 (80.0)	5 (20.0)			4 (16.0)	15 (60.0)	6 (24.0)		
**Somatic disorders**																						
Hypertension	No (409)	287 (70.2)	122 (29.8)	0.2	0.1	299 (73.1)	110 (26.9)	0.3	0.5	43 (10.5)	366 (89.4)	0.7	0.4	327 (79.9)	82 (20.1)	0.8	0.3	74 (18.1)	281 (68.7)	54 (13.2)	62.3	0.0
	Yes (91)	57 (62.6)	34 (37.4)			69 (75.8)	22 (24.2)			7 (7.7)	84 (92.3)			69 (75.8)	22 (24.2)			3 (3.3)	44 (48.4)	44 (48.4)		
Cancer	No (431)	305 (70.8)	126 (29.2)	5.6	0.0	310 (71.9)	121 (28.1)	4.5	0.0	42 (9.7)	389 (90.3)	0.2	0.6	342 (79.4)	89 (20.6)	0.0	0.8	70 (16.2)	286 (66.4)	75 (17.4)	10.0	0.0
	Yes (69)	39 (56.5)	30 (43.5)			58 (84.1)	11 (15.9)			8 (11.6)	61 (88.4)			54 (78.3)	15 (21.7)			7 (10.1)	39 (56.5)	23 (33.3)		
Diabetes	No (441)	312 (70.6)	129 (29.4)	6.6	0.0	318 (72.1)	123 (27.9)	4.3	0.0	49 (11.1)	392 (88.9)	5.1	0.0	356 (80.7)	85 (19.3)	5.3	0	77 (17.5)	305 (69.1)	59 (13.4)	94.1	0.0
	Yes (59)	32 (54.2)	27 (45.8)			50 (84.6)	9 (15.3)			1 (1.7)	58 (98.3)			40 (67.8)	19 (32.2)			1 (0.5)	20 (32.9)	39 (66.1)		
Acid Peptic Disease (APD)	No (444)	310 (69.8)	134 (30.2)	1.9	0.1	334 (75.2)	110 (24.8)	5.4	0.0	45 (10.1)	399 (89.9)	0.1	0.7	352 (79.4)	92 (20.7)	0.0	0.9	73 (16.5)	290 (65.3)	81 (18.2)	6.6	0.0
	Yes (56)	34 (60.7)	22 (39.3)			34 (60.7)	22 (39.3)			5 (8.9)	51 (91.1)			44 (78.6)	12 (21.4)			4 (7.1)	35 (62.5)	17 (30.4)		
Thyroid	No (463)	325 (70.2)	138 (29.8)	5.7	0.0	337 (72.8)	126 (27.2)	2.1	0.1	50 (10.8)	413 (89.2)	4.4	0.0	367 (79.3)	96 (20.7)	0.0	0.8	76 (16.4)	308 (66.6)	79 (17.1)	27.0	0.0
	Yes (37)	19 (51.4)	18 (48.6)			31 (83.8)	6 (16.2)			1 (1.3)	36 (98.7)			29 (78.4)	8 (21.6)			1 (2.7)	17 (45.9)	19 (51.4)		

Hospitalization was highest for cancer (43%), thyroid (42%), APD (39%), followed by hypertension (37%), and diabetes (29%). For all the conditions except cancer and thyroid, private providers were the more preferred. Minimum stay duration in the hospitals for the above-mentioned conditions for all patients was more than 4 days in case of hospitalization. It was observed that they were taking 1–5 medications on an average.

Psychiatric conditions such as mood disorders, schizophrenia, schizotypal and delusional disorders, and mental, behavioral disorders due to psychoactive substance use were significantly associated with polypharmacy. Neurotic, stress-related and somatoform disorders and mental, behavioral disorders due to psychoactive substance use were significantly associated with duration of stay and hospitalization in a facility. Under physical conditions, hypertension, cancer, diabetes, APD, and thyroid disease were significantly associated with polypharmacy. Having cancer, diabetes, and APD was significantly related to visits to public healthcare providers.

Table 6 explains the variations in OOPE (in INR) by morbidity conditions among the patients. As per the varied psychiatric conditions, the mean OOPE was calculated concerning the presence of certain psychiatric (if not one then it may represent the presence of other psychiatric conditions), physical, and combined (presence of both) conditions. It was observed that among all these patients, the highest OOPE was incurred on the laboratory and diagnostic tests, followed by medicines and travel expenditure. Overall, mean OOPE was highest for mood disorders (INR 7004.2) followed by mental/behavioral disorders due to psychoactive substance use (INR 5922.2), and organic, including symptomatic/mental disorders/behavioral syndromes/physiological disturbances/physical factors/mental retardation (INR 5866.0). Further, in patients with physical morbidities, it was observed that the highest OOPE was incurred on the laboratory and diagnostic tests, followed by medicines and transport cost. Overall highest mean OOPE was recorded for hypertension (INR 12340.3), followed by diabetes (INR 12252.5), and APD (INR 10371.1).

**Table 6:** Variations in OOPE (In INR) by morbidity conditions among the sampled population.

**Disease conditions**	**Medicines**			**Travel related expenses**			**Laboratory/diagnostics**			**Total expenditure**		
	**Mean (95% CI)**			**Mean (95% CI)**			**Mean (95% CI)**			**Mean (95% CI)**		
**Psychiatric conditions**												
Mood disorders	1156.3	959.9	1352.8	746.4	582.3	910.6	2656.7	1941.8	3371.6	4559.5	3699.6	5419.3
	1381.6	1199	1564.1	1300.3	935.3	1665.3	4322.4	2939.7	5705	7004.2	5449	8559.5
Schizophrenia, schizotypal and delusional disorders	1315.8	1164.2	1467.3	1141.5	892.3	1390.8	3821	2874.7	4767.3	6278.3	5203.3	7353.4
	1090.7	804.6	1376.7	572.9	394.3	751.4	2230.2	1162.3	3298.1	3893.7	2596.2	5191.1
Neurotic, stress-related and somatoform disorders	1284.5	1138.2	1430.8	1059.3	818.1	1300.4	3698.1	2749.9	4646.4	6041.9	4965.5	7118.3
	1215.2	885.2	1545.2	900.3	579.5	1221.1	2711.8	1673	3750.6	4827.3	3519.5	6135.2
Mental/behavioral disorders due to psychoactive substance use	1282.3	1143	1421.7	973.5	772.7	1174.3	3535.1	2698	4372.2	5790.9	4836.1	6745.8
	1137.7	639.6	1635.8	1652.3	591.6	2712.9	3132.3	1011.6	5252.9	5922.2	3343.6	8500.8
Organic, including symptomatic/mental disorders/behavioral syndromes/physiological disturbances/physical factors/Mental retardation	1270.6	1132.1	1409.1	1045.6	832.7	1258.5	3549.8	2727.1	4372.5	5866	4925.8	6806.3
	1273.2	737.6	1808.8	689.6	247.8	1131.4	2610.8	566	4655.6	4573.6	2093.7	7053.5
**Somatic disorders**												
Hypertension	1002	879.3	1124.6	848.5	644.3	1052.8	2496.1	1855.4	3136.7	4346.5	3597.8	5095.2
	2478.9	2072	2885.8	1833.5	1219.2	2447.8	8027.9	4947.6	11108.3	12340.3	9007.4	15673.2
Cancer	1221.2	1079.9	1362.5	1008.7	783.7	1233.6	3429.3	2589.8	4268.9	5659.2	4705	6613.3
	1580.4	1177.1	1983.7	1147.3	697.1	1597.5	3962.2	1686.6	6237.7	6689.9	4001.4	9378.4
Diabetes	1048	924.4	1171.5	904.4	713.2	1095.6	2985.9	2291	3680.8	4938.3	4120.4	5756.3
	2936	2448.7	3423.3	1949.8	1010.7	2889	7366.6	3269.8	11463.4	12252.5	7985.7	16519.2
APD	1212.5	1075.6	1349.5	953	739	1167	3059.5	2269.7	3849.4	5225.1	4320.4	6129.7
	1732.4	1238.4	2226.4	1621.1	986.8	2255.3	7017.7	3935	10100.4	10371.1	6913.6	13828.6
Thyroid	1190.2	1058.1	1322.2	1012.2	800	1224.3	3383.3	2558.2	4208.5	5585.7	4643.8	6527.5
	2279.5	1605.5	2953.4	1223.2	499.6	1946.9	4998.6	2398.3	7598.9	8501.3	5500	11502.6
**Multiple conditions (both psychiatric and somatic)**												
Mood disorders and Hypertension	1140.6	1004.9	1276.3	880.4	686.7	1074.2	2793.1	2170.9	3415.3	4814.1	4079.6	5548.7
	2416.7	1974.4	2859	2325.1	1352	3298.2	9751.6	4566.3	14936.9	14493.3	8970.6	20016.1
Other psychiatric conditions and Hypertension	1175.1	1045.1	1305.1	956.4	758.4	1154.3	3363.5	2526.8	4200.2	5495	4544.2	6445.8
	2370.8	1691.2	3050.3	1849.3	733.3	2965.2	5105.2	3024.7	7185.8	9325.2	6805.6	11844.8
Mood disorders and Diabetes	1144.3	1011.6	1276.9	935.2	747.6	1122.8	3116	2434.1	3797.9	5195.5	4389.6	6001.4
	2766.1	2250.6	3281.5	2122.6	779.8	3465.3	8075.4	2096.7	14054.1	12964	6774.5	19153.5
Other psychiatric conditions and Diabetes	1183.8	1055.8	1311.8	998.2	789.8	1206.6	3400.3	2590.2	4210.4	5582.3	4658.3	6506.2
	3254.8	2271.3	4238.3	1702.9	795.2	2610.6	5842.3	2653.8	9030.9	10800	7246.1	14353.8
Mood disorders and Cancer	1227.8	1087	1368.6	1005.2	791.4	1219.1	3402.5	2594.9	4210.1	5635.5	4712.8	6558.2
	1778.6	1370.5	2186.7	1294.4	644.3	1944.4	4689.5	1358.2	8020.8	7762.4	3935.8	11589.1
Other psychiatric conditions and Cancer	1258.5	1124	1393.1	1024	810.7	1237.2	3513.7	2693.4	4334.1	5796.2	4863.2	6729.3
	1449.5	716.7	2182.3	1083.9	452.4	1715.4	3343.7	521.1	6166.3	5877.2	2312.8	9441.5
Mood disorders and APD	1241.7	1103.5	1380	985.4	778.2	1192.6	3231.9	2469.8	3994.1	5459.1	4586.3	6331.9
	1779.1	1254	2304.2	1770.1	780.5	2759.8	8248.9	2548.3	13949.5	11798.1	5386.7	18209.6
Other psychiatric conditions and APD	1238.5	1105.2	1371.8	980.6	770.5	1190.6	3349.7	2527	4172.4	5568.8	4628.3	6509.2
	1743.1	980.2	2506	1718.3	923.3	2513.3	5742.8	3203.3	8282.2	9204.1	6353.1	12055.2
Mood disorders and Thyroid	1202.2	1069	1335.4	1013	803.7	1222.2	3370.3	2558.3	4182.3	5585.5	4657.2	6513.7
	2384.1	1666	3102.2	1268.6	386	2151.3	5655.9	2426	8885.7	9308.6	5683	12934.2
Other psychiatric conditions and Thyroid	1260.5	1127.1	1393.9	1027.3	821.1	1233.5	3517.3	2717.2	4317.4	5805.1	4890.9	6719.3
	1900	104	3696	1058.8	−9	2126.5	2616	256.1	4975.9	5574.8	1375.2	9774.3

When both psychiatric (into two categories i.e., mood disorders and other) and physical conditions combined, it was observed that laboratory tests and diagnostics have contributed the highest share in the overall OOPE. Furthermore, in the case where mood disorders were coexisting with other physical conditions, it was observed that mood disorders along with hypertension was resulting in the highest mean OOPE (INR 14493.3), followed by mood disorders and diabetes (INR 12964), and mood disorders and APD (INR 11798). The maximum share of OOPE for both psychiatric and physical morbidities was attributed to laboratory investigations/diagnostics.

Multimorbidity was constantly associated with higher mean OOPE (INR 12219) for the patients (Annexure 1) as compared to only psychiatric conditions (INR 4414). It was observed that increasing age (60+), working as casual laborers, and others (Housewives and students), currently married, non-Hindu were positively associated with the higher OOPE among the patients with multiple morbidities. In case of only psychiatric conditions, being elderly, having education up to higher and senior secondary level, being in other occupations (housewives and students) category, females and residence in rural areas were resulting in higher levels of OOPE.

It was found that the age of patients (especially 60+), having a general caste, visit to private providers, and polypharmacy were associated with a higher OOPE on medicines (Table 7). However, travel-related expenditure was higher for patient living in rural area (β= 0.6; 95% CI: 0.2, 1.1), who were non-Hindus (β= 1.0; 95% CI: −0.1, 2.1) or graduates and above educated (β= 0.9; 95% CI: 0.1, 1.7). Furthermore, we found that increasing age, public and private providers, being married, or taking multiple drugs were more likely to be associated with higher OOPE on diagnostic services. Overall, the age of the patients, consultations and visits to public and private providers, and polypharmacy were the leading contributors to higher OOPE among the patients.

**Table 7:** Factors influencing Out of pocket expenses in people living with psychiatric conditions.

**Covariates**	**Medicine related OOPE**			**Travel related OOPE**			**Diagnostic related expenses**			**Total OOPE**		
	**Coefficient**	**CI (95%)**		**Coefficient**	**CI (95%)**		**Coefficient**	**CI (95%)**		**Coefficient**	**CI (95%)**	
**Age (Ref. 18–39 Years)**												
40–59	0.3	0.0	0.7	0.3	−0.2	0.9	0.7	0.2	1.3	0.5	0.2	0.9
60+	0.6	0.1	1.1	0.5	−0.3	1.2	1.0	0.3	1.8	0.8	0.3	1.3
**Gender (Ref. Male)**												
Female	−0.0	−0.3	0.3	−0.3	−0.7	0.2	−0.2	−0.6	0.3	−0.1	−0.5	0.2
**Residence (Ref. Urban)**												
Rural	−0.2	−0.5	0.1	0.6	0.2	1.1	0.1	−0.5	0.6	0.1	−0.2	0.3
**Caste (Ref. SC/ST)**												
General	0.4	−0.0	0.8	0.5	−0.2	1.2	0.0	−0.7	0.8	0.2	−0.3	0.6
**Religion (Ref. Hindu)**												
Non-Hindu	0.8	−0.2	1.2	1.0	−0.1	2.1	−0.2	−1.3	0.9	0.4	−0.4	1.1
**Marital status(Ref. Unmarried/divorced/widowed)**												
Currently married	−0.2	−0.5	0.2	0.3	−0.2	0.8	0.6	0.0	1.1	0.3	−0.1	0.7
**Education (Ref. Not educated)**												
Up to primary	−0.3	−0.8	0.2	−0.1	−0.9	0.7	−0.6	−1.4	0.3	−0.2	−0.8	0.4
Up to higher/senior secondary	−0.1	−0.6	0.4	0.3	−0.4	1.1	−0.0	−0.9	0.8	0.1	−0.5	0.6
Graduation and above	−0.1	−0.6	0.4	0.9	0.1	1.7	0.5	−0.4	1.4	0.5	−0.1	1.0
**Occupation (Ref. Regular/salaried/business)**												
Casual laborers	0.3	−0.2	0.7	−0.3	−0.9	0.4	0.1	−0.6	0.8	0.1	−0.4	0.5
Others	−0.1	−0.4	0.3	−0.2	−0.7	0.3	0.3	−0.3	0.8	0.1	−0.3	0.5
Unemployment	0.0	−0.4	0.4	−0.0	−0.6	0.6	0.1	−0.6	0.7	0.0	−0.4	0.5
**Social security (Ref. No)**												
Yes	−0.2	−0.6	0.1	−0.4	−0.9	0.1	−0.1	−0.7	0.5	−0.1	−0.5	0.3
**Hospitalization in the facility (Ref. Not hospitalized)**												
Hospitalized	0.2	−0.3	0.6	−0.4	−1.1	0.4	0.3	−0.5	1.1	0.2	−0.3	0.7
**Visit public providers (Ref. Never)**												
One or more times	0.3	−0.0	0.6	0.4	−0.1	0.9	0.9	0.4	1.5	0.6	0.2	0.9
**Visit private providers (Ref. Never)**												
One or more times	0.6	0.1	1.1	−0.1	−0.8	0.7	0.9	0.2	1.7	0.5	−0.0	1.0
**Duration of stay in the facility (0-3 days)**												
4+ days	0.1	−0.4	0.6	0.3	−0.4	1.1	0.8	−0.1	1.6	0.4	−0.1	0.9
**Polypharmacy (Ref. No medicines)**												
1–5 medicines	2.3	1.9	2.7	0.2	−0.4	0.9	0.8	0.1	1.4	0.7	0.3	1.2
6+ medicines	2.6	2.1	3.2	0.5	−0.4	1.4	1.3	0.4	2.1	1.2	0.6	1.8

*OPPE, Out of pocket expenses; SC, Scheduled Castes; ST, Scheduled Tribes*.

## Discussion

Multimorbidity, the concurrent presence of multiple chronic conditions in individuals, is a major problem in clinical care and associated with inferior outcomes (2–4). Additionally, the presence of psychiatric morbidity, such as depression, anxiety, has further negative impact on clinical outcomes (14–17). However, most health systems especially the specialized care facilities are generally configured for management of domain specific diseases instead of multimorbidity in LMICs (1–5). The concept of multimorbidity is hither to unexplored; and yet to be well-integrated into general medical care and research, the knowledge deficit being more pronounced in psychiatric practice (18–20). Our study, first in India aimed to describe the prevalence and correlates of multimorbidity in patients attending psychiatric care settings, assess the differential impact of multimorbidity on health care utilization and medical expenditure, and determine the interaction effect of psychiatric illness on this association.

### Prevalence

Half of the participants had multimorbidity with 45% having more than two conditions indicating higher magnitude in patients attending psychiatric clinics than primary care. The estimated prevalence of multimorbidity among general practice patient population in LMICs has been found to be ranging from 17.5 to 37.3% (48). Our previous study in 40 primary care clinics in India had found one third outpatients to be multimorbid (18). The higher prevalence in our study sample might be for two prime reasons. First, unlike primary care, the probability of a condition getting diagnosed is higher in a specialist hospital with the availability of laboratory facility and different clinical specialties (13, 18, 38). Secondly, the presence of psychiatric illness usually potentiates the common risk factors resulting in accumulation of concordant morbidities (18–20). Given the increased health system contact, the subsequent conditions do also get diagnosed earlier (18–20). It is worthwhile to note that, the observed prevalence may be under-reported, since, the patients requiring hospitalization might not have participated in the study. Future study should consider including both outpatients and inpatients to have a better estimation.

Worldwide, mood disorders and schizophrenia are the most leading psychiatric conditions (16, 17, 26, 30). National Mental Health Survey (NMHS), in India has also observed the prevalence of mood disorders to be the highest followed by schizophrenia and other disorders (30), which is similar to the case load prevalence in our patients. Within physical conditions, the frequency of hypertension and diabetes was in alignment with primary care, while the prevalence of cancer was found to be much higher compared to primary care (13, 18, 27–29). There are two possible explanations. Because of the psychological ill-health of cancer patients, they might be referred by the oncologists for psychiatric consultation as it's a specialist hospital (29–33). Additionally, few studies have documented the disconnect between primary care and oncology practice in India entailing that once diagnosed, cancer patients often do not consult primary care even for their routine health needs (19, 42).

The psychiatric morbidities estimates between primary care vs. specialist outpatient in high-income countries are comparable with no gross difference (24, 31, 33). Contrarily, we found the prevalence of psychiatric illness to be much higher in a specialist care setting reflecting the weak link between primary care and psychiatric services, a phenomenon endemic to most of the LMICs (10, 13). Thus, on one hand, multimorbidity impacts adversely mental health while at the same time the mental illnesses do not get diagnosed in primary care thus creating dual challenge.

### Correlates

It is concerning to notice that the mean age of study participants was below 40 years with more than half (55%) being in the age group of 18–39 years followed by 40–49 years (32%). Of those with multimorbidity, 42.1% were aged below 60 years, indicating a premature onset of psychiatric illness and concomitant multimorbidity compared to available reports from middle and high income countries (14–17). Overall, the frequency of psychiatric as well as physical morbidities (except hypertension and diabetes) was higher in younger patients (18–39 years). Even though, the median age of psychiatric disorders is tilted toward younger age group, the co-presence of psychiatric and physical conditions at a young age is inundated with challenges (30). Apart from unfavorable treatments outcomes and poor prognosis, it impairs quality of life and heightens the probability of premature mortality (49). Furthermore, being multimorbid at a younger age increases the length and volume of health care use, putting perennial three-dimensional stress on individuals, families and health services providers alike (12–16).

We found the number of conditions to be rising with increasing age. This is expected since age is an established risk factor for NCD (27). Thus, over a period of time, the projected number of individuals having psychiatric multimorbidity could be substantial. This not only will strain the individuals and health systems, but decelerate the economic productivity of the society (27, 50). Being the largest user of health care resources at the most socially active or economically productive age group, has a profound impact on national growth. As research into mental-physical multimorbidity in youth is still largely unexplored in India (13), future study should specifically address this knowledge gap. Adopting a longitudinal study to investigate factors contributing to premature onset of psychiatric multimorbidity in younger age population could provide insights for early life interventions to promote young people health.

We did not find any significant difference between male and female with respect to multimorbidity. The evidence on the association of gender with multimorbidity has been inconclusive with conflicting reports (46, 50). Though variations in the gender based prevalence of specific psychiatric or physical conditions are observed, but when it pertains to multimorbidity (simple count of conditions) there is no distinct difference (46). Moreover, similar to other studies, we could not find any supporting evidence on the role of educational attainment, marital status and caste in multimorbidity (18–22).

### Social Deprivation and Equity

Overall, half of the study participants belonged to rural areas. Traditionally believed to be an urban phenomenon, NCDs are no longer confined to high socioeconomic strata and pervaded rural geography (30, 31). Our findings are in alignment with available reports on the obliterating rural-urban divide in prevalence of chronic physical and psychiatric conditions (46).

Research from both high- and middle-income countries reveal that the onset of multimorbidity is one decade earlier in socially deprived population with higher rates in younger age groups being more frequent (4, 5, 7, 10, 12, 13, 13). Additionally, these individuals under increased social deprivation often experience increased levels of depressive symptoms and lower levels of self-reported well-being (10, 13). These salient findings reinforces the need for revitalizing the primary care in hard to reach areas to respond ably both chronic physical and mental illness management (50–53).

With regard to source of livelihood and occupation, casual laborers (daily wagers) were having highest level of multimorbidity followed by those who had no employment. To add on, three fourth of the patients were not having any social security scheme while one third did not have any independent source of income. A study in UK has reported that being income poor was significantly associated with worse mental health (OR 1.63; CI 1.28–2.09) while marginally with physical health (OR 1.34; CI 1.00–1.80) after adjustments (54). As, income and livelihood are acknowledged influencers of physical illness and psychological health, the higher level of mental illness among individuals working in unstable employment could be partly for income insecurity. Thus, the level of deprivation not only affects the volume but also the type of multimorbidity that people experience and treatment outcomes (54). Furthermore, a greater mix of mental and physical problems is seen as deprivation increases, which means increased clinical complexity and the need for holistic person centered care (15, 24, 31, 55). Hence, current universal health coverage programme i.e., National Health Assurance Programme (NHAP) in India may consider expanding to informal or unorganized employment sectors for work place-based health care along with adequate financial protection (35).

### Healthcare Utilization

Around three fourth of the respondents never visited any public healthcare system rather sought care from the private facilities irrespective of the age group. The National Sample Survey report echoes observation akin to this (46). Private sectors are a key stakeholder in delivering health services comprising routine, emergency, medical, surgical and critical care (6, 18, 46). About 90 percent of 40–59 years' patients have recorded highest visits to the private clinics demonstrating a tilting of younger psychiatric patients toward non-public health system. Few studies in India have reported that younger age group try to seek care from private providers for reasons like perceived better quality of care, hospital infrastructure than public care system and shorter waiting time (28). It is cited that to avoid loss of wages, and income for sick leave patients in working age group prefer to visit a private care provider at their own convenience (28, 46). Within psychiatric morbidities, mood disorders had highest number of consultation and hospital visit and those having schizophrenia, schizotypal and delusional disorders, have availed multiple consultations too. Mood disorders comprising both manic and depressive episodes are recognized for their unpredictable clinical course requiring frequent monitoring and multiple consultations for stabilization (52). Within physical conditions, private providers were preferred for cancer and thyroid only. Having diabetes, and APD was significantly related to visits to public healthcare provider's. In our prior primary care study, older people were found to favor public health system more for their chronic physical conditions (3). This contrary yet interesting finding reveals that irrespective of age groups, multimorbidity patients prefer to visit private health care when one of the condition is psychiatric. One of the explanations could be the need for privacy and confidentiality as psychiatric illness is still perceived to be a taboo in the society with low cultural acceptance (30, 31). Moreover, the perceived ease of emergency admission and expectation of personalized care appear to be another influencing factor (30, 31). Future research should explore the care navigating mosaic for psychiatric illness and understand the decision-making process, encompassing social, cultural, economic, and health system dimensions.

Patients with psychiatric disorders often receive a multiple medication regimen (polypharmacy) as pharmacological treatment is a cornerstone in psychiatry care (55). Almost eighty percent were taking 5–8 drugs daily (51, 52, 55). More than two-third patients of 18–39 years were being prescribed 1–5 medicines while half of the elderly (60+ years) were on more than six medicines. Though, it's common to have polypharmacy in elderly, the higher prevalence of the same in a relatively younger patient population is worrisome (51, 55). Across psychiatric illness spectrum, mood disorders accounted for highest number (>5 medicines per day) followed by schizophrenia, schizotypal and delusional disorders. Given its' dual spectrum, mood disorders require multiple overlapping medications to provide immediate amelioration in acute mania while waiting for the long-term effect of another mood stabilizer; and to add an antidepressant to a mood stabilizer when a bipolar patient develops a depressive episode (52).

Strangely, patients with hypertension, cancer, diabetes, APD, and thyroid were also taking higher number of medications than routine practice (6, 27). Apparently, the presence of psychiatric illness increases the number of prescription medicines in these patients, multiple doctors often prescribing medications thus adding to the pre-existing treatment burden. Polypharmacy, though often appropriate for psychiatric multimorbidity can increase the risk of drug-drug and drug-disease interactions, medication errors and poor treatment adherence, and cause unnecessary burden for families as they manage complex medication schedules and worsening of health outcomes especially in young age group (51, 52, 55). This can be equally problematic for elderly patients who are at an elevated risk of treatment complications and need to be de-prescribed judiciously (51, 52, 55). Eliciting the opinions of different clinical specialists including psychiatrist on the perceived relevance of polypharmacy is necessary toward designing personalized therapeutic management for multimorbidity in psychiatric patients.

Around one-third of mood disorder patients had frequent hospital admissions with in-hospital stay averaging 4 days. Neurotic, and somatoform and psychoactive substance use disorders were second in row. Mood disorders are complex characterized by alternating or overlapping episodes of mania and depression with profound disruptive effect on patients. Many times, they need urgent intervention by psychiatrist and hence necessitate frequent emergency hospital admission (56). We found multimorbidity to increase the odds of hospital admissions, similar to international literature (50). The rate or number of unplanned admissions is found to be significantly higher in low resource settings putting a stress both on patients and health system (56). In India, given the limited psychiatric care providers, availing regular psychiatric consultation is difficult and continuity of care is a challenge. Thus, many of these patients might be seeking care only when there is an acute aggravation or upon emergency (56). Compared to psychiatric illness, the proportion of patients availing hospitalization was lesser for patients with physical morbidities. Conversely, the mean duration of stay was higher (more than 4 days) for these patients particularly cancer (43%), thyroid (42%), APD (39%), followed by hypertension (37%), and diabetes (29%). It is noteworthy that hypertension, diabetes and APD when they occur alone require much lower in-hospital stays and hospital admissions (27, 56). The higher hospital visits and in-patient stay here could be attributed to their coexistence with psychiatric morbidity, one mutually exacerbating the other. Studies from UK and Canada have demonstrated that addition of each physical illness was associated with a greater increase in the odds of frequent visits to the emergency department for people with mental disorders (57).

Across the four major groups of psychiatric conditions, mood disorders had significantly highest level of healthcare use. The volume of care demands become multiplicative when they have physical morbidities like cancer, thyroid disease, diabetes. Thus, the combination of mental-physical multimorbidity poses compounding challenges for the patients encompassing polypharmacy, multiple consultations, frequent hospitalization, repeated laboratory investigation as well as navigating multiple specialities. Prior research suggests that psychiatric multimorbidity patients especially younger age group are at increased risk of suicidal ideation fuelled by perpetual treatment burden and psychological ill-health and thus, merits the careful attention of treating clinicians (58). An in-depth understanding of the lived experiences and challenges of these patients, a sub-sample of the present cohort, could provide key insights.

### Healthcare Expenditure

Multimorbidity was consistently associated with higher (three times more) mean OOPE (INR 12219) as compared to only psychiatric conditions (INR 4414) in our study patients. Within multimorbidity, OOPE was significantly greater in elderly age group (60+), those working as casual laborers (daily wagers) or those without any regular source of income (housewives and students. Obviously, these group of patients if not covered under any financial or social security, have to rely on others to meet the high health care expenditure accrued. Such superimposed burden of perpetual OOPE might lead to chronic depletion of financial resources in turn placing households at risk for impoverishment, which is noteworthy to consider. Literatures suggest that coexisting mental disorders interact with physical morbidity to increase the odds of frequent visits to the emergency department, and consequential OOPE, the risk being exacerbated by socioeconomic deprivation ((27–29)). Furthermore, residing in rural areas and being female were additional correlates of higher levels of OOPE in patients having singular psychiatric condition (monomorbidity). The sub-optimal availability or low penetration of mental health care to rural locations with limited number of trained psychiatrist could be responsible for OOPE in rural areas. At present both state and central government are establishing health and wellness centers (HWC) to deliver integrated care for NCD (59). These settings can be the fulcrums for holistic health promotion and act as interface between patients and specialist care. Moreover, in view of limited training on geriatric health and paucity of competence in geriatric mental health, building the capacity of primary care providers in geriatric mental health should be a step in this direction.

Disaggregated analysis reveals that within multimorbidity, overall mean OOPE was highest for mood disorders (INR 7004.2) followed by mental/behavioral disorders due to psychoactive substance use (INR 5922.2). Previous studies have demonstrated that mood disorders being associated with polypharmacy and hospital visits incur substantial OOPE (52). It is stated that the characteristic episodic and bipolar nature of the illness involves acute management, which adds to the cost (24). Within physical morbidities, highest mean OOPE was recorded for hypertension (INR 12340.3), followed by diabetes (INR 12252.5). Furthermore, mood disorders when coexisting with hypertension resulted in the highest mean OOPE (INR 14493.3), followed by mood disorders and diabetes (INR 12964). An assessment of metabolic and cardio-vascular morbidities at the time of any hospitalization or consultation is a routine practice in psychiatry toward deciding treatment options. Moreover, many of the anti-psychiatric medications especially for mood disorders are known to have effect on metabolic and vascular parameters and require regular monitoring (52). Our findings in concurrence with observations from other high income countries reiterate that addition of physical condition with psychiatric morbidity, or “multimorbidity” leads to substantially higher healthcare resource use than individual psychiatric or physical conditions; the resultant effect being multiplicative than additive. This not only unveils the challenges of a psychiatrist but also other clinical disciplines while managing such patients. An exploration of how these clinicians manage psychiatric multimorbidity patients would illuminate the therapeutic conflicts and help in preparing a standard care protocol.

Among patients with multimorbidity as well as those having singular or monomorbidity, highest OOPE was incurred for the laboratory and diagnostic tests, followed by medicines and travel expenditure. Irrespective of psychiatric and physical morbidities, laboratory tests and diagnostics have accounted for the largest share of OOPE thus being major contributor. First, often, the psychiatric and neurological conditions have symptoms overlapping making it difficult for the treating clinician to differentiate. Hence these patients may have been prescribed cost—involving neuro—imaging investigations, more so, during emergency admission, to support precise diagnosis of neuropsychiatric disorders (55). These patients being at risk for drug-drug interaction also need periodic monitoring of laboratory parameters. One more factor accounting for high OOPE could be repeated laboratory investigations in different facilities at frequent interval for the same illness since majority patients have sought care from multiple providers before arriving at the definitive specialist setting. Future study should explore the need and rationality of prescribing such frequent and repeated laboratory investigations by the physicians, especially when they are cost intensive. Any future health insurance or financial protection scheme for catastrophic health expenditure should not only take into account multimorbidity but to minimize the large cost of laboratory investigations/ diagnostics. Having low cost point of care diagnostics should be a policy priority under the universal health coverage. At present, the state government's free access to laboratory investigations, is limited to public care facilities (28). Looking at the volume and demand for private sector, it might be prudent to expand the scope of the state's program to non-public sector and consider subsidize these diagnostic cost to reduce the OOPE by the patients. Having a unique patient code or clinical electronic record system with empanelled quality assured designated laboratories could be thought of as a strategy in long term to avoid duplication of investigations and optimize costs and resources.

It was found that being aged, visit to private providers, and polypharmacy were associated with a higher OOPE on medicines. Polypharmacy is known for its adverse effects more in elderly apart from leading to higher expenditure and suboptimal adherence. Thus, aged patients who are on multiple medications and belong to lowest income range appear to be the most disadvantaged. In polypharmacy, the unit cost of medicines for mood disorder is more. One reason could be that to manage acute episodes in mood disorders, mostly parenteral medications are preferred the raised cost. Supplementary medications to augment the nutritional status is commonly used during acute phases where more hospital consultation or admission is required.

Amongst the components of OOPE, though travel-related expenditure contributed to the smallest share, yet, it is notably higher for patients living in rural area (β=0.6; 95% CI: 0.2, 1.1), compared to urban. This is expected as people residing in rural areas need to visit the definitive care facility for every consultation, which increases commensurately with the count and type of condition as well as the nature and duration of treatment (56). The added cost for transport might also predispose to discontinuation of treatment or disrupted care. To address the dual disadvantage of access and affordability, technology based psychiatric consultation could be explored as a complementary approach. Involvement of primary care practitioners to deliver basic psychiatric care follow ups with mentoring of a psychiatrist will help in ensuring seamless care while reducing the burden of OOPE on the patient.

Substantial epidemiological evidence has shown that social and economic deprivations are associated with poorer health outcomes particularly with a greater prevalence of colorectal cancer, cardiac disease, musculoskeletal pain, as well as increased mortality rates (28, 50). Objective measures of relative deprivation and income inequality are seen to be especially linked to poor mental health (28). The fact that life expectancy for those with major mental illness is 20 years less for men and 15 years less for women is a stark statistic of mental health inequality globally (28, 50). Surprisingly, little research has examined whether measures of relative deprivation and inequity is contributing to poorer health outcomes in multimorbidity more so when one of the concomitant condition is mental. Our study findings, in concurrence with prior observations, reinforce that individuals living in deprived locations, with no regular source of income and in the absence of financial protection, might be disproportionately affected by the negative consequences of inequity and inequality and be more at risk for multimorbidity with poorer health outcomes (28). Within a national health care system universal coverage, this association likely reflects unmet need due (at least in part) to the continuing problem of the “inverse care law” (60). This reiterates the need for providing universal health coverage with ample financial protection or more intensified in these socially, geographically and economically deprived locations to prevent impoverishment and promote holistic health.

## Strengths and Limitations

Our findings should be interpreted bearing in mind few limitations. Since the present study investigated multimorbidity among the patient attending a single psychiatric care setting, those who stayed at home and did not seek psychiatric consultation, institutionalized and hospitalized people might have been missed and could not be included and bears restricted scope for generalisability. In order to garner a better understanding of the country level magnitude, we envisage to undertake multi-clinic based study with adequate geographic representation and sample size in recent future. Although results presented herein has shown the size, patterns, and determinants of multimorbidity burden, the cross-sectional nature of the study precluded us to draw inferences about the underlying causal relationships between co-occurring chronic diseases and their impact on the trajectories of clinical progression and outcomes. The joint evolutionary course of multimorbidity might be captured with refined probabilistic models following a cohort study design (61). Nonetheless, in the absence of any comprehensive, population-based studies, our paper provides the maiden evidence on multimorbidity in the context of psychiatric care settings and its impact on health services costs and health care use. The findings offer valuable insights for incorporating multimorbidity into clinical decision-making and health services design, and delineate critical knowledge gaps and research priorities to advance the care of psychiatric patients with multimorbidity, especially those of advanced and younger age (62).

## Implications for Practice, Policy and Research

Multimorbidity is more common in patients attending psychiatric clinics when compared to primary care. For clinicians involved in psychiatric care for an aging patient population who have multiple diseases, multimorbidity is the rule not the exception. The interaction of different diseases and the impact they have on important clinical outcomes, such as physical function, quality of life and mortality, should all be considered by the psychiatrist. Since suicidality has been shown to be strongly associated and a potential outcome with multimorbidity, clinicians/physicians should consider assessing suicidal thoughts or ideation in these patients while designing therapeutic regimen. The ubiquitous presence of polypharmacy in psychiatric multimorbidity insinuates the need to consider the therapeutic benefits and side effects to ensure a beneficial risk-benefit ratio while prescribing multiple-regimen. Given the potential of treatment conflict and drug-drug interaction, a harmonious coordination between clinicians and psychiatrists is a must for managing psychiatric multimorbidity.

As younger age group constitute a major share for whom multimorbidity happens earlier, future studies should examine potential interventions to improve the mental health of young patients especially those residing in communities with greater social deprivation. Further, an assessment of mental health literacy in adolescent age group and design prevention strategies and the allocation of resources to best support youth and families. The early onset of psychiatric multimorbidity in Indian demography merits longitudinal studies that more definitively clarify the temporality of physical and mental disorder onset and the extent to which both physical and mental health influences ultimate multimorbidity treatment outcomes. Few cohort studies could additionally explore the care seeking, care challenges and coping relating to psychiatric multimorbidity with special focus on self-care and resilience building.

The direct effect of multimorbidity on the health care utilization and medical expenditure is evident being disproportionately high when mood disorders and hypertension coexist in a patient. As the most vulnerable to impoverishment are those not having any organized job, not covered by any financial protection and from the lowest income quintile, strong advocacy to expand the present scope of the National Health Assurance Program (NHAP) to add emergency hospitalization for acute episodes of mood disorders into the existing list of operative or treatment procedures is warranted (35). Minimizing the cost incurred from laboratory investigations and diagnostic procedures through subsidizing or co-financing appears to be important in order to prevent catastrophic expenditure. Considering the affinity of psychiatric multimorbidity patients to private care despite being costlier, inclusion of private sector for psychiatric care for low-income patients under the ambit of NHAP may be explored.

Whilst multimorbidity is more common in older people, the strategies to control chronic conditions in India should not be limited to older adults only since majority of psychiatric multimorbidity belong to younger age group too. The designated Health and Wellness Centers (HWCs) could be leveraged to horizontally integrate the national non-communicable diseases and mental health program and geriatric care program (59). Toward this, strengthening the primary care settings and creating a “community of practice” for multimorbidity should be a key strategy.

## Conclusion

In conclusion, our findings contribute to the evidence that in persons with psychiatric illness, multimorbidity occur early in life necessitating early detection and treatment initiation. The higher presence of OOPE in the lowest quintiles and deprived ones reflect the continuing existence of the “inverse care law” and the need for whole system changes to enhance the effectiveness of primary care for patients with psychiatric multimorbidity in deprived areas. Action is required to redress this mismatch of need and service provision for psychiatric patients with multimorbidity if health inequalities are to be narrowed rather than widened by primary care. In view of limited training on geriatric health and paucity of competence in geriatric mental health, capacity building of primary care providers in geriatric mental health should be a step forward.

Multimorbidity in mental health may be a relatively new concept for psychiatry but is likely to become increasingly important in the future particularly for India. Given the central role of mental illness within the multimorbidity continuum, it is critical that psychiatrists, primary care practitioners, researchers and policy makers consultatively deliberate on how best to develop and evaluate services that will improve physical, psychological and social outcomes for these patients. Seamless integration of mental and physical health services toward family-centered models of care delivery are to be prioritized.

## Data Availability Statement

The raw data supporting the conclusions is with the corresponding author and can be made available on reasonable request and prior approval.

## Ethics Statement

The studies involving human participants were reviewed and approved by The Institutional Ethics Committee of KIMS, Bhubaneswar, Odisha (Vide no. KIMS/KIIT/IEC/204/2018 dated 14.12.2018). The patients/participants provided their written informed consent to participate in this study.

## Author Contributions

SP, PM, and KS conceived the study. SP, PM, KS, and RCD designed the tool of the study. PM and MS collected data for the study. RD, RA, MH, and SP analyzed the data. All authors were involved in drafting the manuscript, reviewing, and approved the final manuscript.

## Conflict of Interest

The authors declare that the research was conducted in the absence of any commercial or financial relationships that could be construed as a potential conflict of interest.

## Appendix

**Annexure 1:** Socio-economic differentials in OOPE (In INR) for the sampled population with no and multi-morbidity conditions.

**Covariates**	**Only psychiatric**								**Multi-morbidity**							
	**Medicines**		**Travel**		**Lab/diagnostics**		**Total expenditure**		**Medicines**		**Travel**		**Lab/diagnostics**		**Total expenditure**	
	**Mean**	**SE**	**Mean**	**SE**	**Mean**	**SE**	**Mean**	**SE**	**Mean**	**SE**	**Mean**	**SE**	**Mean**	**SE**	**Mean**	**SE**
Age																
18–39	696.47	67.26	596.58	84.45	1269.15	238.37	2562.20	301.76	1333.67	152.99	1172.81	274.08	3797.81	946.35	6304.29	1084.20
40–59	886.75	142.43	375.50	93.98	1195.17	518.04	2457.42	548.28	1782.77	153.47	1807.65	362.31	6435.17	1191.67	10025.59	1346.81
60+	646.15	188.32	903.08	256.96	2561.54	566.51	4110.77	559.46	2704.35	326.59	1482.00	302.03	7707.69	2088.20	11894.04	2202.09
Gender																
Female	798.92	86.18	595.52	124.40	1647.16	375.18	3041.59	461.39	1923.14	174.13	1438.65	248.12	5526.65	942.74	8888.44	1061.50
Male	688.10	82.10	528.25	58.41	1034.18	223.51	2250.52	248.66	1684.69	146.58	1551.81	293.10	5842.51	1162.64	9079.01	1289.22
Residence																
Urban	714.87	86.03	417.92	104.48	1058.32	251.43	2191.11	376.85	1926.95	160.23	1406.07	307.08	5980.84	1152.66	9313.86	1309.07
Rural	759.85	82.30	676.20	81.99	1533.28	325.77	2969.34	339.37	1642.98	157.60	1610.76	196.23	5313.27	870.75	8567.02	918.95
Caste																
SC/ST	573.65	124.85	517.57	142.11	1155.14	375.51	2246.35	420.79	1621.48	494.54	1252.59	349.69	6071.85	2730.81	8945.93	2940.13
General	768.33	66.22	566.74	72.99	1347.00	240.07	2682.07	287.75	1823.86	112.78	1525.62	211.36	5640.52	774.99	8990.01	869.63
Religion																
Hindu	743.36	60.76	548.15	67.06	1347.55	219.28	2639.07	261.86	1736.47	107.89	1453.97	199.22	5745.56	776.43	8936.01	861.26
Non-Hindu	636.67	296.80	862.22	300.98	543.33	250.69	2042.22	400.66	2828.67	832.22	2156.67	722.71	4771.33	2935.75	9756.67	3590.85
Marital status																
Unmarried/divorced/widowed	594.81	60.23	590.83	99.49	1183.79	278.04	2369.42	299.46	1754.20	253.40	1080.00	389.44	3636.10	921.78	6470.31	1263.97
Currently married	840.92	91.12	537.48	87.24	1413.06	303.44	2791.46	375.61	1816.77	126.69	1624.68	220.65	6320.66	935.22	9762.12	1017.24
Education																
Not educated	680.91	230.11	571.36	154.43	724.09	284.17	1976.36	408.11	1651.07	276.47	800.86	239.00	4693.57	1212.90	7145.50	1231.65
Up to primary	768.06	101.57	467.96	75.86	1312.04	388.00	2548.06	427.15	1752.30	315.74	1179.46	268.24	6332.16	2839.67	9263.92	2914.67
Up to high/senior secondary	934.42	140.42	581.28	141.78	1939.87	561.55	3455.58	669.64	2066.76	247.81	1351.06	277.73	4839.52	1079.98	8257.33	1210.85
Graduation & above	587.92	68.52	584.41	109.79	971.49	212.51	2143.81	262.28	1706.13	156.04	1838.66	357.71	6190.41	1132.07	9735.20	1322.39
Occupation																
Regular/salaried/business	762.00	126.92	568.30	123.18	931.00	339.89	2261.30	419.51	1702.08	194.18	1588.10	351.15	4017.14	906.42	7307.32	1118.77
Casual laborers	813.20	164.41	277.20	98.50	444.00	150.53	1534.40	283.11	1893.02	304.10	1619.65	379.04	7713.49	2951.84	11226.16	3162.63
Others	764.00	93.19	577.25	110.98	1708.67	393.45	3049.92	468.01	1858.90	195.43	1367.06	291.60	6286.38	1107.77	9512.35	1258.96
Unemployment	632.18	115.19	640.91	124.78	1217.45	283.55	2490.55	325.59	1750.38	255.72	1503.10	628.79	5094.29	1524.63	8347.76	1688.21
Social security																
No	758.97	65.81	579.44	90.65	1155.24	196.35	2493.65	275.82	1848.01	127.66	1613.93	231.72	5923.42	892.63	9385.36	1000.85
Yes	698.19	122.83	517.00	70.30	1665.75	513.99	2880.94	531.73	1613.27	248.31	1012.94	231.86	4717.76	1107.56	7343.96	1143.40
Total	1340.00	512.77	381.43	83.40	2692.86	1453.93	4414.29	1488.08	2592.42	217.92	1717.86	254.88	7909.04	1617.21	12219.31	1667.32

## Abbreviations

AOR: Adjusted odds ratio
APD: Acid peptic disease
APL: Above poverty line
BPL: Below poverty line
CHC: Community health center
CI: Confidence interval
IRR: Incidence rate ratio
LMIC: Low- and medium-income countries
NCD: Non communicable disease
PHQ: Patient health questionnaire.
